# Immunology in Africa

**DOI:** 10.1111/tmi.12599

**Published:** 2015-10-12

**Authors:** Stephen Cose, Bernard Bagaya, Barbara Nerima, Moses Joloba, Andrew Kambugu, Robert Tweyongyere, David W. Dunne, Edward Mbidde, Pontiano Kaleebu, Alison M. Elliott

**Affiliations:** ^1^ MRC/UVRI Uganda Research Unit on AIDSEntebbeUganda; ^2^ Clinical Research DepartmentLondon School of Hygiene & Tropical MedicineLondonUK; ^3^ Department of Medical MicrobiologyMakerere University College of Health SciencesKampalaUganda; ^4^ Uganda Virus Research InstituteEntebbeUganda; ^5^ Infectious Diseases InstituteMakerere University College of Health SciencesKampalaUganda; ^6^ Department of Veterinary Pharmacy, Clinical and Comparative MedicineMakerere UniversityKampalaUganda; ^7^ Department of PathologyCambridge UniversityCambridgeUK

**Keywords:** immunology, Africa

## Abstract

Africa is a continent with a large burden of both infectious and non‐communicable diseases. If we are to move forward as a continent, we need to equip our growing cadre of exceptional young scientists with the skills needed to tackle the diseases endemic to this continent. For this, immunology is among the key disciplines. Africans should be empowered to study and understand the diseases that affect them, and to perform their cutting‐edge research in their country of origin. This requires a multifaceted approach, with buy‐in from funders, overseas partners and perhaps, most important of all, African governments themselves.

## Introduction

In many areas, Africa has been described as the ‘Continent of the Future’. Yet despite this optimism, there remains a large disease burden throughout the continent, exacerbated by poor health systems and a lack of expertise in critical areas 1, 2, 3. A good scientific base, in which African scientists can tackle the diseases that affect their communities, is critical to Africa's development 4, 5. External scientific expertise and funding are not sustainable resources to address Africa's problems. Identifying the scientific needs and supporting the best young Africans to develop the tools necessary for prevention, diagnosis and treatment of endemic diseases should be a priority for all African nations.

Understanding the diseases that affect us requires a multidisciplinary approach. The Holy Grail for many infectious disease researchers is to find a successful vaccine that prevents us from contracting the disease, or from spreading it to others. Historically, most of our successful vaccines have been developed empirically. Finding a successful vaccine for the many remaining diseases will require a more rational approach to vaccine design, and to do this, we need to understand the immunology behind the disease or infection. Immunological tools are also important in the development of diagnostics (rapid tests for HIV and malaria are examples), and sometimes in therapy. This report discusses the need to further develop scientific research in Africa from an immunological perspective, identifies key barriers to generating a critical mass of immunologists who can tackle the burden of today's diseases in Africa and describes some of the steps we have already taken towards this goal.

## Immunological expertise is needed in Africa

Immunology is a relatively new discipline, and even newer to the continent of Africa. The lack of immunological expertise across the continent is highlighted by the continent's immunological publication outputs (Table 1). South Africa, the most developed country, is ranked first in Africa but with an output comparable to Poland, 16th among European countries (Table 1). Kenya and Uganda (ranked 2nd and 3rd within Africa) fall well below the top 20 in terms of European countries (Table 2), although even European science lags behind that of the USA in terms of immunological output and impact (Table 3). Nigeria, Africa's wealthiest nation 6, is ranked only 12th among the African nations (Table 1).

**Table 1:** Immunology output of the top 20 African countries (1996–2013). Output ranked on H index, a measure of impact from the research output. Data obtained from SCImago Journal & Country Rank (http://www.scimagojr.com), accessed 13 April 2015. Data are from the Immunology and Microbiology section from SCImago

Rank	Country	Citable documents	Citations	Citations per document	H index
1	South Africa	7453	134758	20.74	121
2	Kenya	2843	60503	24.26	90
3	Uganda	1357	26664	24.87	70
4	Tanzania	1426	27283	22.63	65
5	Senegal	1071	18570	18.48	62
6	Gambia	548	14073	25.54	60
7	Malawi	602	13620	27.57	56
8	Cameroon	1162	17186	16.76	54
9	Zambia	559	11407	24.38	54
10	Ghana	912	15900	19.67	52
11	Côte d'Ivoire	599	10870	18.34	52
12	Nigeria	3686	25983	9.93	51
13	Burkina Faso	793	14002	21.34	51
14	Tunisia	2022	19852	15.25	49
15	Ethiopia	851	11957	18.11	48
16	Zimbabwe	559	9818	17.95	45
17	Gabon	470	7988	17.82	45
18	Morocco	885	9210	12.48	41
19	Sudan	635	8414	16.75	41
20	Mali	387	6720	21.72	40

**Table 2:** Immunology output of the top 20 European countries (1996–2013). Output ranked on H index, a measure of impact from the research output. Data obtained from SCImago Journal & Country Rank (http://www.scimagojr.com), accessed 13 April 2015. Data are from the Immunology and Microbiology section from SCImago

Rank	Country	Citable documents	Citations	Citations per document	H index
1	United Kingdom	79201	2590073	31.88	359
2	Germany	67651	2147660	32.51	353
3	France	54499	1608328	29.03	302
4	Italy	35717	974429	28.41	276
5	Netherlands	28289	962503	34.91	274
6	Switzerland	20141	776740	40.09	271
7	Sweden	19546	589792	30.33	220
8	Spain	30699	703785	24.23	211
9	Belgium	14857	440608	31.3	202
10	Denmark	12282	359960	30.21	188
11	Austria	9562	298973	33.09	185
12	Finland	7726	223800	29.18	158
13	Ireland	5342	157267	33.64	153
14	Norway	6494	185200	29.92	150
15	Greece	4736	105160	24.58	110
16	Russian Federation	12641	113805	9.11	108
17	Poland	9450	109371	12.5	107
18	Hungary	3983	78230	20.53	105
19	Portugal	5699	104312	22.17	104
20	Czech Republic	6514	92618	15.13	99

**Table 3:** Immunology output of the USA (1996–2013). Data obtained from SCImago Journal & Country Rank (http://www.scimagojr.com), accessed 13 April 2015. Data are from the Immunology and Microbiology section from SCImago

Rank	Country	Citable documents	Citations	Citations per document	H index
1	United States	274951	9621324	35.75	589

Clearly, there is a need to build a stronger immunological base across the whole of Africa, so that African scientists can compete for funding at the same level as their European and USA counterparts. But what are the issues and bottlenecks that are preventing young Africans from gaining the immunological knowledge required to establish themselves as leaders in their own field?

## Issues and bottlenecks

### Theoretical Immunology

Taught courses are essential for any discipline, and immunology is no exception. However, outside of South Africa, there is no university department devoted exclusively to teaching and research in immunology. As a consequence, medical and veterinary schools across Africa teach only very basic immunology as a topic within the disciplines of Microbiology or Pathology. Recognising this need, our Capacity Building Programme (www.muii.org.ug) set out to build a short course in immunology to meet the growing needs of the continent. This course has been very successful, and we have trained over 300 young scientists in both theoretical and practical immunology, with extremely good feedback (Box 1). Our programme supports undergraduates, postgraduates, postdocs and university lecturers. Building on the success of our Programme, the Department of Medical Microbiology within the College of Health Sciences at Makerere University (Uganda) has recently proposed a new Department of Immunology. This Department, when inaugurated, will house the MSc in Immunology and Clinical Microbiology, which has recently been developed (http://mbl.mak.ac.ug).

Box 1 **Sample feedback from the MUII Immunology in the Tropics course**

The well‐organised training delivered in an all inclusive participatory way was highly motivating to me as a researcher. Lectures, laboratory‐based practicals and discussion on hot topics guided by prominent faculty members were insightful and provided the much needed current knowledge on immunology of malaria and helminths. Research tools acquired from the iTROP course were helpful in securing a collaborative grant for the purpose of undertaking seroprevalence survey for Dengue and Chikungunya viruses.

I partly dedicate my recent achievement of a postdoctoral grant award by the Consortium for National Health Research (CNHR) to iTROP course work for its motivation, provision of current knowledge and immunology practicals provided.

If anybody has benefited from the training it is me. I have attended two courses, and the training has contributed a lot to my profession:

It will contribute towards my promotion

Improvement of my area of specialisation

Obtained the latest facts and research avenues in areas such as immunology of malaria and helminths

As a lecturer, I have improved my delivery to my students, both undergraduates and postgraduates.

The materials obtained from the training are quite resourceful for teaching the latest immunology and are my day to day guide to my teaching notes.

It was a wonderful experience, and made me love more a career in Immunology. Over the course, I was able to understand the basic and advanced concepts in immunology, current and advanced techniques used in Immunology assays through lectures, seminars and journal clubs sessions. The lively debate at the help sessions, the audience Q&A following a stimulating lecture all cultivated my own enthusiasm, research directions and development as a scientist.

The quality time we spent interacting with world‐class immunologists through dedicated revision time and social events was priceless. The many hours spent with each of us listening to them, and talking with them about our goals and plans for the future, were incredibly rewarding.

I learnt so much in both trainings and have been able to use most of what I learnt in writing a grant application focusing on malaria and helminth infections, and also in designing and optimising my laboratory assays. The training also enabled me interact with trainers and trainees who were an inspiration to me.

This is to express my gratitude for again another opportunity to attend the Immunology in the Tropics course.

Besides the knowledge I acquired through the lectures, my highlight was that I learnt to know the reason behind each step in a protocol. This has indeed set me in a position to probe for better understanding of the reasons behind each protocol step, in which way getting better informed and hitting the road to being a better laboratory technologist/scientist.

This has not left me the same.

Despite our recent success, there are still challenges in delivering appropriate taught content because there are few trained immunologists to give expert and in‐depth foundation lectures. We have sought to address this by bringing together, in Uganda, the leading health research organisation (The Uganda Virus Research Institute; www.uvri.go.ug) with the leading University (Makerere University; www.mak.ac.ug). This has allowed the teaching programmes within the University to be enriched by the expert knowledge within the leading research institution. In addition, we have equipped several campus sites with teleconference equipment to broaden the reach of local research seminars and visiting scientist seminars. This two‐way interaction enables us to reach out to a wider interest group who may like to attend the seminar remotely, because they are otherwise unable to attend due to scheduling conflicts or travel time. Finally, we will soon begin adding expert international guest lectures to taught courses at Makerere University, to account for the lack of expertise in certain areas. We will begin piloting this with colleagues at Cambridge University, through the Cambridge‐Africa Programme (http://www.cambridge-africa.cam.ac.uk).

In our experience, students acquire a deeper understanding of concepts and cutting‐edge advances in the field if the person teaching them has extensive experience of the technology, technique or discipline. Didactic teaching, which is often done where immunological expertise is lacking and where classes are large, is an uninspiring way of communicating and inhibits interest in the field. In our experience, students respond best to interactive teaching, a method of approach that perhaps requires further implementation in many universities on the African continent.

These challenges are partially mitigated by the many online resources available for those with an interest in this field. Companies such as Becton Dickinson (www.bdbiosciences.com) and ThermoFisher Scientific (www.thermofisher.com; incorporating Life Technologies and Invitrogen among other companies) offer very good tutorials and online content for the budding immunologist. In addition, the Federation of African Immunological Societies (www.faisafrica.com), and more broadly the African Academy of Sciences (www.aasciences.org) websites can point interested parties to upcoming immunological conferences and courses, and are well worth visiting. Finally, Purdue University (www.cyto.purdue.edu) offers a curated discussion board specifically to address issues on flow cytometry, the essential tool of the immunologist. This is not an exhaustive list of online help for the immunologist, but these are among the good websites available for the student and lecturer alike to visit and gain insight and inspiration into the field of immunology.

### Animal models

In most areas throughout Africa, researchers are devoted to understanding the human and animal diseases that are endemic throughout Africa, and this is as it should be. But an area that is often overlooked is the laboratory animal models that have contributed a large amount of knowledge to any field of immunology. Mouse immunology (for example) and human immunology are, of course, very different. But understanding an animal model, regardless of how imperfect the model is, can help in understanding the mechanism of the response in humans. A novel finding in humans can lead to new hypotheses, but determining the mechanism behind the finding is often not achievable in human studies. The novel human finding can be backed up with animal studies to identify the mechanism, and conversely, mechanistic studies developed in animals can be tested in human cohorts to see whether similar responses occur.

Little mouse immunology is taught because there is simply not the expertise in Africa to teach the animal models. This needs a deep understanding of the animal model, and this cannot be achieved through reading papers or textbooks alone. Hands‐on experience is needed, as well as active research on animal models. Of course, most countries in Africa do not have the resources to sustain specific pathogen‐free (SPF) animal research facilities to explore these animal models. At some point, this needs to happen in Centres of Excellence across Africa, but the cost of setting up these facilities in the absence of a critical mass of within‐continent researchers is prohibitively expensive and limiting. Currently, the International Livestock Research Institute (www.ilri.org) and the Institute of Primate Research (www.primateresearch.org) provide examples where animal research is being conducted on the continent. But these are few compared with the number of human studies that are ongoing, and perhaps the best opportunity we can give our African researchers is the opportunity to collaborate on research projects involving animal models. Split‐site PhD and postdoctoral studies may be an ideal way to integrate human and animal model immunology research.

### Practical immunology

Immunological studies are increasingly being performed on a daily basis across the whole of Africa, and this is encouraging. However, without the background knowledge of the immunological mechanism(s) behind the test or experiment, researchers (or more often, technicians) are often at a loss as to how to rectify the experiment if it fails. There are many techniques that we, as immunologists, use. Many of them, such as the ELISA, are cheap and easy to perform, and require relatively little training. More and more now, however, complex techniques are being used, such as multicolour flow cytometry, Luminex^®^ technology and immunofluorescent microscopy. We must give our young and enterprising immunologists the capability of understanding the background to the technique, and not just the protocol itself. We should be teaching our cadre of young African scientists to ask ‘how’ and ‘why’. To this end, our Capacity Building Programme (http://www.muii.org.ug), funded by the Wellcome Trust, runs Practical Immunology modules, aimed at teaching participants not only new techniques, but also the mechanisms behind the techniques. This approach has been very successful in empowering young scientists to go back to their home laboratories with renewed enthusiasm and a deeper understanding of the Immunology behind the technique (see Box 1 for an example).

### Journal clubs

Getting a small group of like‐minded individuals to discuss a relevant published paper is an excellent way of integrating and synthesising new concepts in the field. All too often, however, the article that is presented is a small article specific to the area of interest of the individual presenting the paper, and with little challenging material within the actual paper. Challenging articles are avoided because the background immunology is not present to either understand the detail of the experiments that have been performed, or the concept that the paper is putting forward. This is by no means an ‘Africa‐only’ problem. But it should be incumbent upon all of us to encourage our young scientists to present challenging papers.

Of course, choosing the best paper is somewhat subjective and is largely dependent on the skill of the presenter in getting the concepts and reasoning of the authors across to the audience. This is a skill that must also be learnt, and one in which senior colleagues can lead the way. Perhaps the biggest issue then is having senior figures within the room to not only present an article, but also to challenge concepts, to ask questions and to debate in an informative way. Such a figure can inspire and encourage our young African generation to move out of their comfort zone and test themselves with a truly challenging article. During our immunology short courses, our students are given challenging papers to read and present. Our expert international faculty are available to help with interpretation and explanation of concepts and figures, and the students on our courses have found this a truly rewarding experience. Translating this to one's own workplace is the obvious challenge, and we must encourage our senior colleagues to give back their knowledge and experience to the young and enthusiastic researcher.

### Job market

Common to all scientific disciplines is the question every university graduate asks: what now? In Western countries, a vibrant scientific base means that jobs have been relatively easy to find. In Africa, without the strong scientific base, University graduates wishing to find a research post in immunology are often left disappointed. When junior posts do arise, there are often many applications for a single post. This is a good thing as far as an employer goes, as you can often find exceptional talent among the large number of applications. Unfortunately, the lack of available positions also means much of Africa is in an employer's market, which can lead to unethical and abusive practices, such as requiring staff to work overtime without additional pay, or minimising annual leave, for example. More recently, the Wellcome Trust‐funded African Institutions Initiative has provided opportunities for postdoctoral researchers, and more of these initiatives should be encouraged.

It is the experience of many of us that good African researchers are often overwhelmed with teaching and administrative duties when taking up or pursuing their academic goals. This has had the unwanted effect that many Africans are simply unable to keep their research interests alive unless they can secure dedicated protected time from their other academic duties to pursue their research interests. There is also a lack of higher positions (postdoctoral to Lecturer, and above) in low‐ and middle‐income countries. Many graduates want to undertake higher degrees such as an MSc or PhD, but without the positions to go with a higher degree, postgraduates are often forced out of the country to find suitable posts. In addition, obtaining a higher degree often prices them out of the market because of the lack of senior positions. Positions advertising for a technologist often attract MSc graduates, for example. Because we all rely on funding to do our research, often salaries are capped for the position advertised. Although you may get an excellent candidate who holds an MSc, often the salary is such that it is no longer attractive to postgraduates. Without appropriate positions in country, they are forced to look abroad for further work. This contributes to the brain drain of talented Africans heading abroad to pursue academic research and to take up senior positions outside of their home country.

Inviting Biotec companies to establish laboratories on the continent, and investing in young Biotec start‐ups, can also help in preventing the brain drain and opening up new avenues in the job market. Such companies can bring economic benefit in terms of investing in clinical immunology for care and diagnostics. African governments need to recognise the benefit of research and development for the improvement of their own health systems and economy 5.

## Integrating immunology research

A key component for the success of research in Africa will be embedding good immunological studies into basic and clinical research, within the framework of a sound epidemiological background and strong statistical support. Supporting fellows within this framework allows them to identify relevant questions and design the most appropriate study to answer the questions they find most important.

Within our MUII Programme, and working with other partners, we have supported fellows in both basic 7, 8, 9, 10, 11, 12 and clinical 13, 14, 15, 16, 17, 18 research, underpinning this research with good epidemiological design and statistical advice from local and international colleagues. We, and our fellows, have found this an excellent scaffold on which to build the new generation of African science and research.

However, all too often the equipment or expertise to undertake immunological studies in Africa is absent, and samples are sent abroad for well‐funded laboratories to undertake the essential immunological experiments. This has left Africans to undertake the fieldwork and epidemiological studies alone, and this must stop. Minimising the shipment of samples outside of the African country of origin would go some way to ensure that our overseas collaborating partners invest in the country and train our African scientists. Many countries within Africa now require substantial justification to send samples outside of the country. With well‐equipped laboratories now present in many countries, and with the growing expertise, such justification is becoming harder to obtain. There are two notable exceptions where samples should be sent abroad: multisite studies and complex studies requiring specialised resources, such as high‐throughput sequencing. For multisite studies, it can be essential that a single laboratory (but not necessarily a laboratory outside Africa) perform the majority of the work, to ensure standardised protocols and harmony across all sites. For complex studies requiring specialised equipment (such as genomic assays), it may not make economic sense to have expensive equipment in country: indeed, researchers all over the world currently outsource such work to a small number of internationally well‐recognised centres. In such cases, outsourcing to a specialised laboratory that routinely runs the technique of interest is a far cheaper and effective way of undertaking complex research. Here, the critical capacity that must be built for African scientists is the ability to analyse complex ‘big data’.

## Conclusion

So what are the solutions, as we see them? Below we outline a few key areas that we believe need to be strengthened in order for Africans to develop solutions to their own infectious disease burdens.

- establish immunology departments with a mix of expertise, and people with research knowledge to deliver lectures;
- in the short to medium term, harness local expertise from research institutes, and international expertise from collaborating partners, to support the long‐term development of local critical mass;
- develop curricula for structured, taught courses, including techniques used in both human and mouse immunology;
- establish teaching laboratories for the delivery of practical courses to allow students to synthesise the taught material into hands‐on learning;
- integrate epidemiology and statistics into advanced degrees so that students can better understand the causes and associations of disease;
- provide pathways to attract the most talented Africans back to their home country, and long‐term support for them in their country;
- research partnerships between Western and African Universities, as equal partners;
- minimise the transfer of biological samples outside of the country of origin, and encourage Western collaborators to invest in people and technology within the country of origin;
- African Governments to recognise the need and positive impact research and development can have on society and allocate funds, however small, for research and development, in their annual budgets.

We are at a turning point in Africa. Countries are emerging from poverty and the time is now right to consider investing in science, for the continued development and growth of the continent. We need to invest in the young scientific generation, and bring back the many talented Africans who are working outside this continent due to a lack of funding and infrastructure. Africans should be empowered to research and understand the diseases that affect them and to perform their cutting‐edge research in their country of origin. This requires a multifaceted approach, with buy‐in from funders, overseas partners and perhaps, most important of all, African governments themselves.
